# Effect of Experimental Parameters on the Hydrothermal Synthesis of Bi_2_WO_6_ Nanostructures

**DOI:** 10.1186/s11671-016-1413-x

**Published:** 2016-04-12

**Authors:** Ziming Cui, Hua Yang, Bin Wang, Ruishan Li, Xiangxian Wang

**Affiliations:** School of Science, Lanzhou University of Technology, Lanzhou, 730050 People’s Republic of China

**Keywords:** Bi_2_WO_6_, Nanostructures, Hydrothermal synthesis, Morphological tailoring, Photocatalytic properties

## Abstract

Bi_2_WO_6_ nanostructures were synthesized by a hydrothermal route, where the effect of various experimental parameters on the products was investigated. It is demonstrated that the sample morphology and size is highly dependent on the NaOH content (or pH value). At *C*_NaOH_ = 0–0.0175 mol (pH range of 1–4), the prepared samples present flower-like hierarchical microspheres which are constructed from thin nanosheets via the self-assembly process. The size of the hierarchical microspheres exhibits a decreasing trend with increasing the NaOH content, from 7 μm at *C*_NaOH_ = 0 mol to 1.5 μm at *C*_NaOH_ = 0.0175 mol. At *C*_NaOH_ = 0.03–0.0545 mol (pH: 5–9), the prepared samples exhibit irregular flake-like structures, and their size increases with the increase in NaOH content. At *C*_NaOH_ = 0.055–0.05525 mol (pH: 10–11), the prepared samples are composed of uniform sphere-like particles with an average size of 85 nm. Compared to the NaOH content, the reaction temperature and time has a relatively small effect on the product morphology and size. The photocatalytic activity of the samples was evaluated by degrading rhodamine B (RhB) under irradiation of simulated sunlight. Among these samples, the samples composed of flower-like hierarchical microspheres have relatively high photocatalytic activity. In particular, the microspheres prepared at *C*_NaOH_ = 0.01 mol exhibit the highest photocatalytic activity, and the degradation percentage reaches 99 % after 2 h of irradiation.

## Background

Hundreds of thousands of tons of organic dyes are produced annually all over the world, most of which have a complicated polyaromatic structure and are hardly decomposed by self-purification action. The dyes in wastewater must be removed or destroyed to an acceptable level before the dye wastewater is released into the natural environment. Among various wastewater treatment technologies, semiconductor-based photocatalysis allows the use of sunlight, a clean and renewable source of energy, for the destruction of dye pollutants, and is regarded as a green technology to solve the environmental problem. TiO_2_ is one of the most famous photocatalysts, exhibiting powerful capabilities for photocatalytically decomposing all types of organic dyes completely into harmless inorganic substances like CO_2_ and H_2_O [1–3]. However, TiO_2_ has a large bandgap energy of ~3.2 eV and is active only under ultraviolet (UV) irradiation with wavelengths below 390 nm, which hinders its widespread application in industrial practice. It is noted that visible light accounts for a fairly large fraction (~45 %) of the solar energy compared to UV light (~5 %). In order to efficiently make use of sunlight to drive photocatalytic reaction, it is desirable to develop photocatalysts that can be excited by visible light in the solar spectrum. In recent years, much work has been concerned with the photocatalysis of Bi-based oxide semiconductors like BiVO_4_, BiFeO_3_, Bi_2_Fe_4_O_9_, Sr_2_Bi_2_O_5_, CaBi_2_O_4_, and Bi_24_O_31_Cl_10_ [4–9]. These semiconductors have a relatively small bandgap energy that is appropriate for the absorption of visible light, making them promising candidates as efficient visible-light-responsive photocatalysts.

Aurivillius compounds with general formula Bi_2_A_n − 1_B_n_O_3n + 3_ (A = Ca, Sr, Ba, Pb, Na, K, and B = Ti, Nb, Ta, Mo, W, Fe) are known to be another important class of Bi-based semiconductor oxides. This class of oxides has a special layer structure with perovskite-like units (A_n − 1_B_n_O_3n + 1_)^2−^ sandwiched between (Bi_2_O_2_)^2+^ layers, and possesses unique physicochemical properties [10]. Bi_2_WO_6_ is one of the simplest (*n* = 1) members of the Aurivillius oxides, exhibiting numerous interesting properties such as ferroelectricity, piezoelectricity, nonlinear dielectric susceptibility, gas sensitivity to alcohol, thermal conductivity, electrochemistry, and photocatalytic activity [11–17]. In particular, since the discovery of the photocatalytic O_2_ evolution from AgNO_3_ solution and photocatalytic degradation of CHCl_3_ and CH_3_CHO over Bi_2_WO_6_ under visible-light irradiation [16, 17], Bi_2_WO_6_ has been extensively studied as a promising visible-light-driven photocatalyst.

It is known that the photocatalytic activity of a photocatalyst is highly correlated with its morphology and size. Among various nano/micro-fabrication techniques, the hydrothermal route offers an advantage in tailoring the product morphology and size, where the main operating parameters include mineralizer concentration, reaction temperature, reaction time, organic additive, etc. However, there has been little systematic investigation into how these parameters affect the synthesis of Bi_2_WO_6_ crystals though several different morphologies of them have been synthesized based on the hydrothermal route [18–26]. In this work, we undertook a systematic investigation on the effects of NaOH (mineralizer) concentration, reaction temperature, and reaction time on the hydrothermal synthesis of Bi_2_WO_6_ nanostructures. The photocatalytic activity of prepared samples was evaluated by the degradation of rhodamine B (RhB) under simulated-sunlight irradiation.

## Methods

All raw materials and reagents used are of analytical grade without further purification. Further, 0.002 mol of Bi(NO_3_)_3_ · 5H_2_O was dissolved in 20 mL acetic acid solution (2.5 mol∙L^−1^) to form solution A, and 0.001 mol of Na_2_WO_4_ · 2H_2_O was dissolved in 20 mL distilled water to form solution B. The above process was accompanied by a constant magnetic stirring to make the additives dissolve fully. Then, solution B was slowly added to solution A drop by drop under constant magnetic stirring, and immediately a milk-white suspension solution was formed. After another 30 min of stirring, a certain amount of NaOH was added to the suspension solution, which was then filled up to 70 mL by adding distilled water. The resultant solution was transferred and sealed in a stainless steel autoclave with a Teflon liner of 100-mL capacity and submitted to hydrothermal treatment at a certain temperature. After a certain time of reaction, the autoclave was naturally cooled down to room temperature. The resultant yellowish precipitate was collected and washed several times with distilled water and absolute ethanol, and then dried in a thermostat drying oven at 60 °C for 8 h to obtain final Bi_2_WO_6_ particles. By varying the NaOH content, reaction temperature, and reaction time, the effects of these parameters on the synthesis of Bi_2_WO_6_ particles were investigated.

The phase purity of the as-prepared Bi_2_WO_6_ samples was examined by means of X-ray powder diffraction (XRD) with Cu Kα radiation. The particle morphology and microstructure was investigated by a field-emission scanning electron microscope (SEM). The UV-visible diffuse reflectance spectrum was measured using a UV-visible spectrophotometer equipped with an integrating sphere attachment. The Brunauer-Emmett-Teller (BET)-specific surface area of the samples was measured by the N_2_ adsorption–desorption technique on an ASAP2020M system.

The photocatalytic activity of Bi_2_WO_6_ particles was evaluated by the degradation of RhB under simulated-sunlight irradiation from a 200-W xenon lamp at room temperature. RhB was dissolved in distilled water to make a 2-mg∙L^−1^ RhB solution. The photocatalyst loading was 0.1 g in 100 mL of RhB solution. Before illumination, the mixed solution was mildly stirred by a magnetic bar for 1 h in the dark to reach the adsorption–desorption equilibrium of RhB on the photocatalyst particles. During the photocatalysis experiment, the water-jacketed reactor was cooled with a water-cooling system to keep the solution at room temperature. At given irradiation time intervals, a small amount of the reaction solution was sampled for examining the RhB concentration, which was determined by measuring the absorbance of the solution at a fixed wavelength of 554 nm using a UV-visible spectrophotometer. Before the absorbance measurements, the reaction solution was centrifuged at 3000 r∙min^−1^ for 10 min to remove the photocatalyst.

## Results and Discussion

### Effect of NaOH Content at *T* = 200 °C and *t* = 24 h

**Fig. 1:**
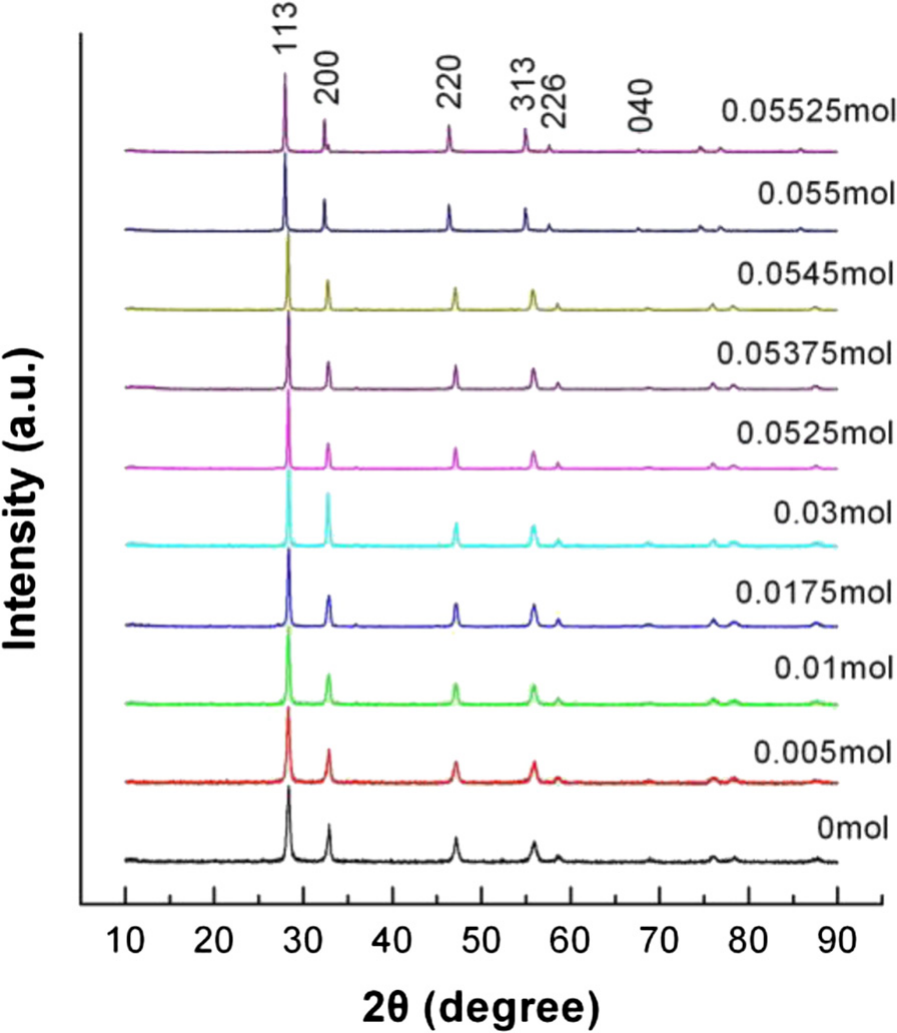
XRD patterns of Bi_2_WO_6_ samples prepared by adding different amounts of NaOH ranging from 0 to 0.05525 mol (correspondingly pH value ranging from 1 to 11), where the hydrothermal reaction temperature and time were fixed at *T* = 200 °C and *t* = 24 h, respectively

Figure 1 shows the XRD patterns of Bi_2_WO_6_ samples prepared by adding different amounts of NaOH ranging from 0 to 0.05525 mol (correspondingly pH value ranging from 1 to 11) while the hydrothermal reaction temperature and time were fixed at *T* = 200 °C and *t* = 24 h, respectively. It is seen that, for all the samples, all the diffraction peaks can be indexed in terms of the orthorhombic Bi_2_WO_6_ phase (PDF card No. 73-2020), and no traces of other impurity phases are detected in the XRD patterns.

**Fig. 2:**
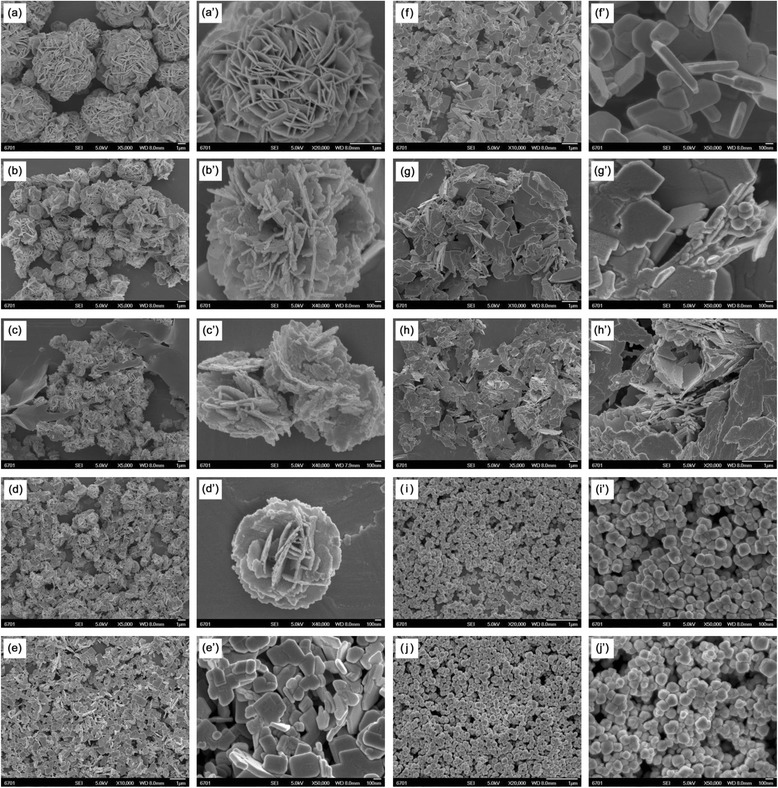
SEM images of Bi_2_WO_6_ samples prepared by adding different amounts of NaOH. **a** *C*_NaOH_ = 0 mol (pH = 1), **b** *C*_NaOH_ = 0.005 mol (pH = 2), **c** *C*_NaOH_ = 0.01 mol (pH = 3), **d** *C*_NaOH_ = 0.0175 mol (pH = 4), **e** *C*_NaOH_ = 0.03 mol (pH = 5), **f** *C*_NaOH_ = 0.0525 mol (pH = 7), **g** *C*_NaOH_ = 0.05375 mol (pH = 8), **h** *C*_NaOH_ = 0.0545 mol (pH = 9), **i** *C*_NaOH_ = 0.055 mol (pH = 10), and **j** *C*_NaOH_ = 0.05525 mol (pH = 11)

Figure 2 shows the SEM images of Bi_2_WO_6_ samples prepared by adding different amounts of NaOH. It is seen that the sample morphology and size is highly dependent on the NaOH concentration (or pH value). When adding small amounts of NaOH in a range of 0–0.0175 mol (pH range of 1–4), the prepared samples mainly consist of flower-like hierarchical structures (see Fig. 2a–d). The size of the flower-like structures exhibits a decreasing trend with increasing the NaOH content, from 7 μm at *C*_NaOH_ = 0 mol to 1.5 μm at *C*_NaOH_ = 0.0175 mol. From Fig. 2e–h, one can see that the samples prepared at the NaOH content ranging from 0.03 to 0.0545 mol (pH value ranging from 5 to 9) exhibit irregular flake-like structures, and their size increases with the increase in NaOH content. The SEM images shown in Fig. 2i, j reveal the synthesis of uniform spherical particles at the NaOH content of 0.055–0.05525 mol (pH range of 10–11). The two samples have a similar average particle size of 85 nm.

### Effect of Reaction Temperature and Time at *C*_NaOH_ = 0 mol (pH = 1)

**Fig. 3:**
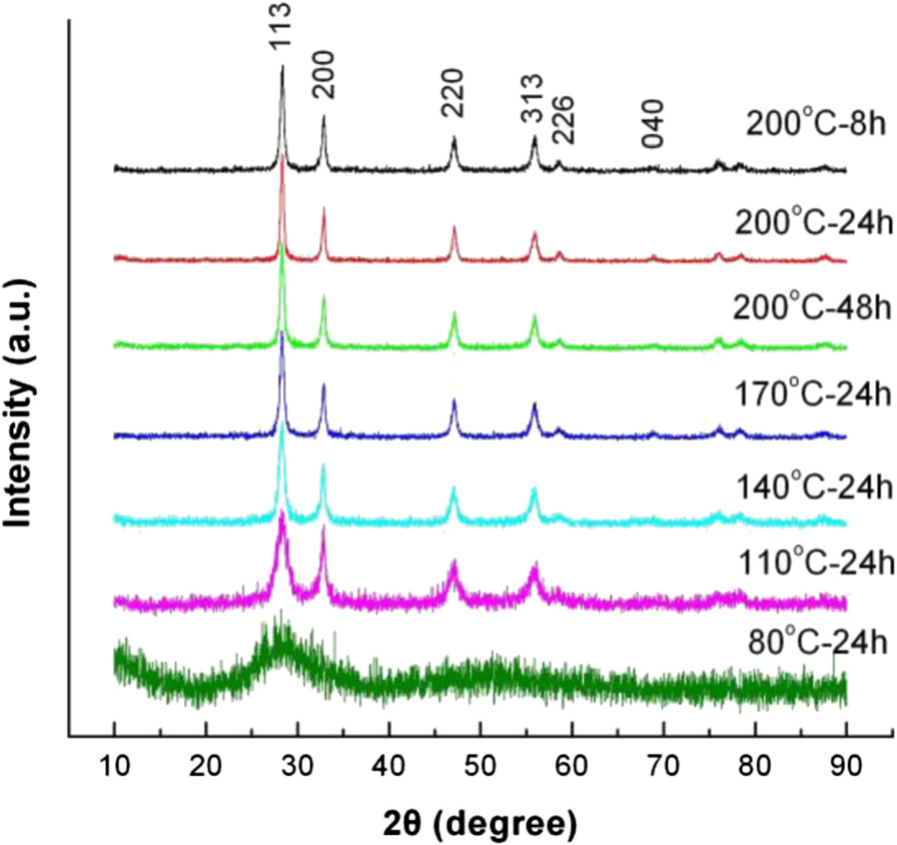
XRD patterns of Bi_2_WO_6_ samples prepared at different reaction temperatures and times without adding NaOH

By fixing the NaOH content separately at *C*_NaOH_ = 0, 0.03, and 0.055 mol, we also investigated the effect of reaction temperature and time on the product morphology and size. Figure 3 shows the XRD patterns of Bi_2_WO_6_ samples prepared at different reaction temperatures and times, where no NaOH was added to the reaction solution, i.e., *C*_NaOH_ = 0 mol. It is seen that the samples prepared at a reaction temperature up to 110 °C crystallize in a pure orthorhombic Bi_2_WO_6_ phase with no traces of second phases. However, when the reaction temperature is below 110 °C, no Bi_2_WO_6_ phase is found to be formed.

**Fig. 4:**
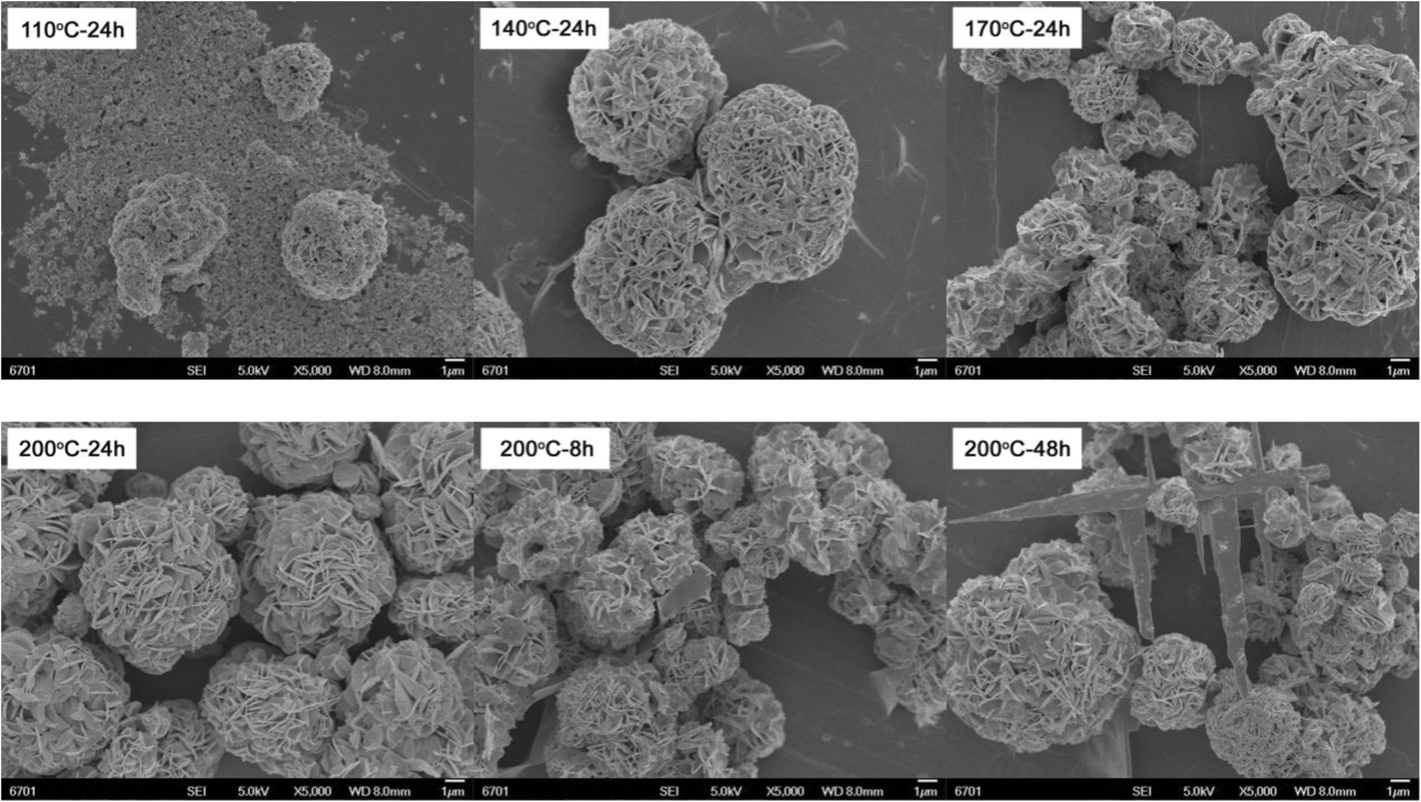
SEM images of Bi_2_WO_6_ samples prepared at different reaction temperatures and times without adding NaOH

Figure 4 shows the SEM images of Bi_2_WO_6_ samples prepared at different reaction temperatures and times without adding NaOH. At the reaction temperature of 110 °C, the prepared sample is mainly composed of irregular particles with a size of several tens of nanometers and minor aggregate microspheres constructed from the nanoparticles. When the temperature is increased above 140 °C, flower-like microspheres are prepared, and these microspheres are constructed from thin nanosheets via the hierarchical self-assembly process. In addition, it is seen that the sample prepared at *T* = 200 °C and *t* = 24 h exhibits relatively more uniform flower-like structures.

### Effect of Reaction Temperature and Time at *C*_NaOH_ = 0.03 mol (pH = 5)

**Fig. 5:**
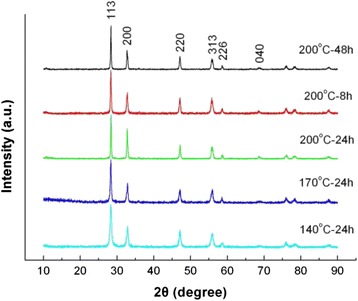
XRD patterns of Bi_2_WO_6_ samples prepared at different reaction temperatures and times with *C*_NaOH_ = 0.03 mol

**Fig. 6:**
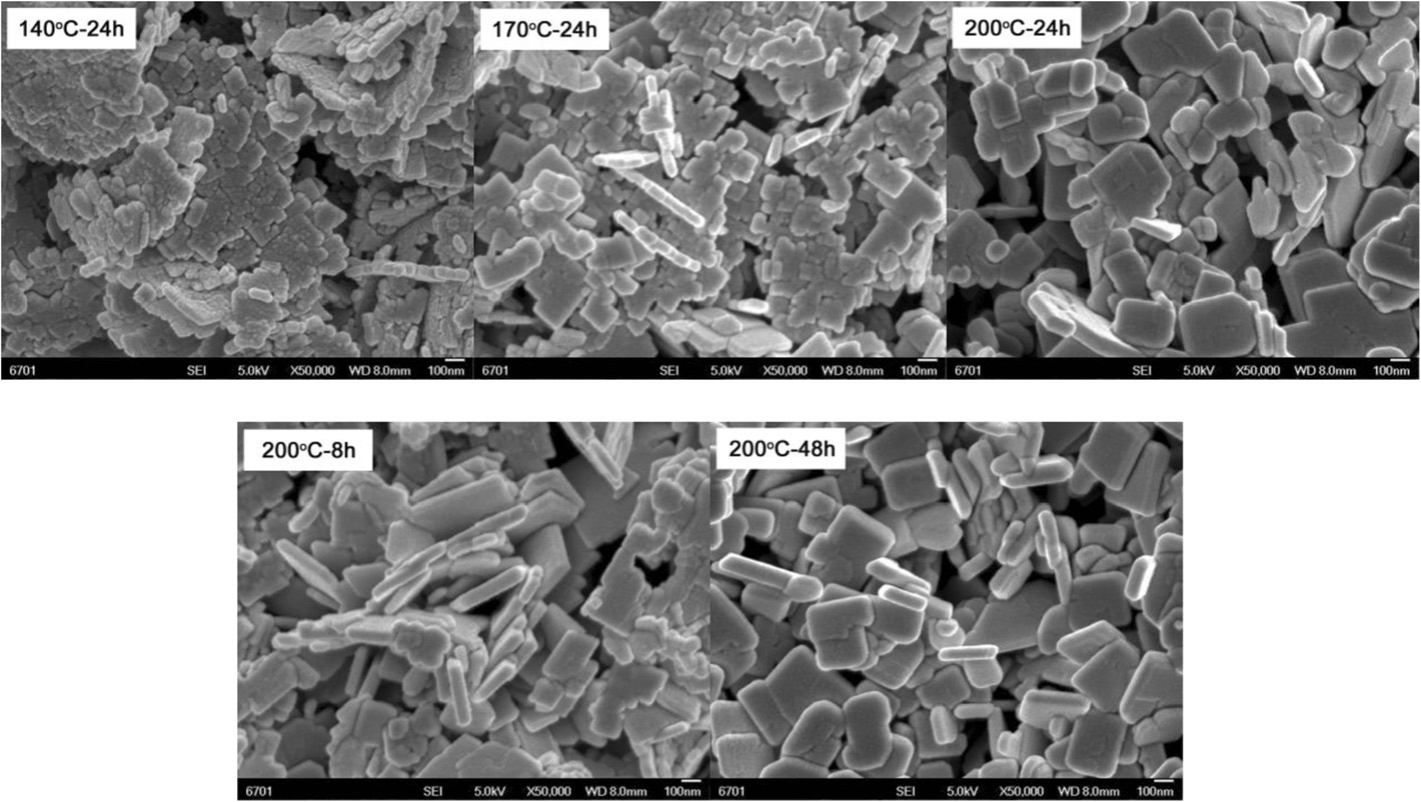
SEM images of Bi_2_WO_6_ samples prepared at different reaction temperatures and times with *C*_NaOH_ = 0.03 mol

Figure 5 shows the XRD patterns of Bi_2_WO_6_ samples prepared at different reaction temperatures and times with *C*_NaOH_ = 0.03 mol, revealing that single-phase Bi_2_WO_6_ samples are synthesized at the reaction temperature of 140–200 °C and reaction time of 8–48 h. Figure 6 shows the SEM images of the prepared Bi_2_WO_6_ samples, from which one can see that all the samples exhibit irregular flake-like structures. With increasing the reaction temperature, the flakes become gradually larger and thicker; however, the reaction time has almost no effect on the morphology and size of the flakes.

### Effect of Reaction Temperature and Time at *C*_NaOH_ = 0.055 mol (pH = 10)

**Fig. 7:**
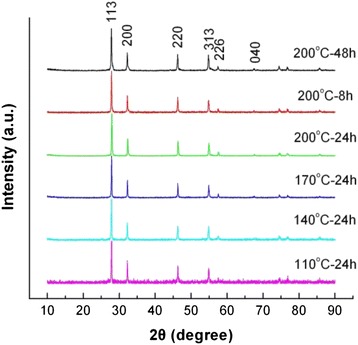
XRD patterns of Bi_2_WO_6_ samples prepared at different reaction temperatures and times with *C*_NaOH_ = 0.055 mol

**Fig. 8:**
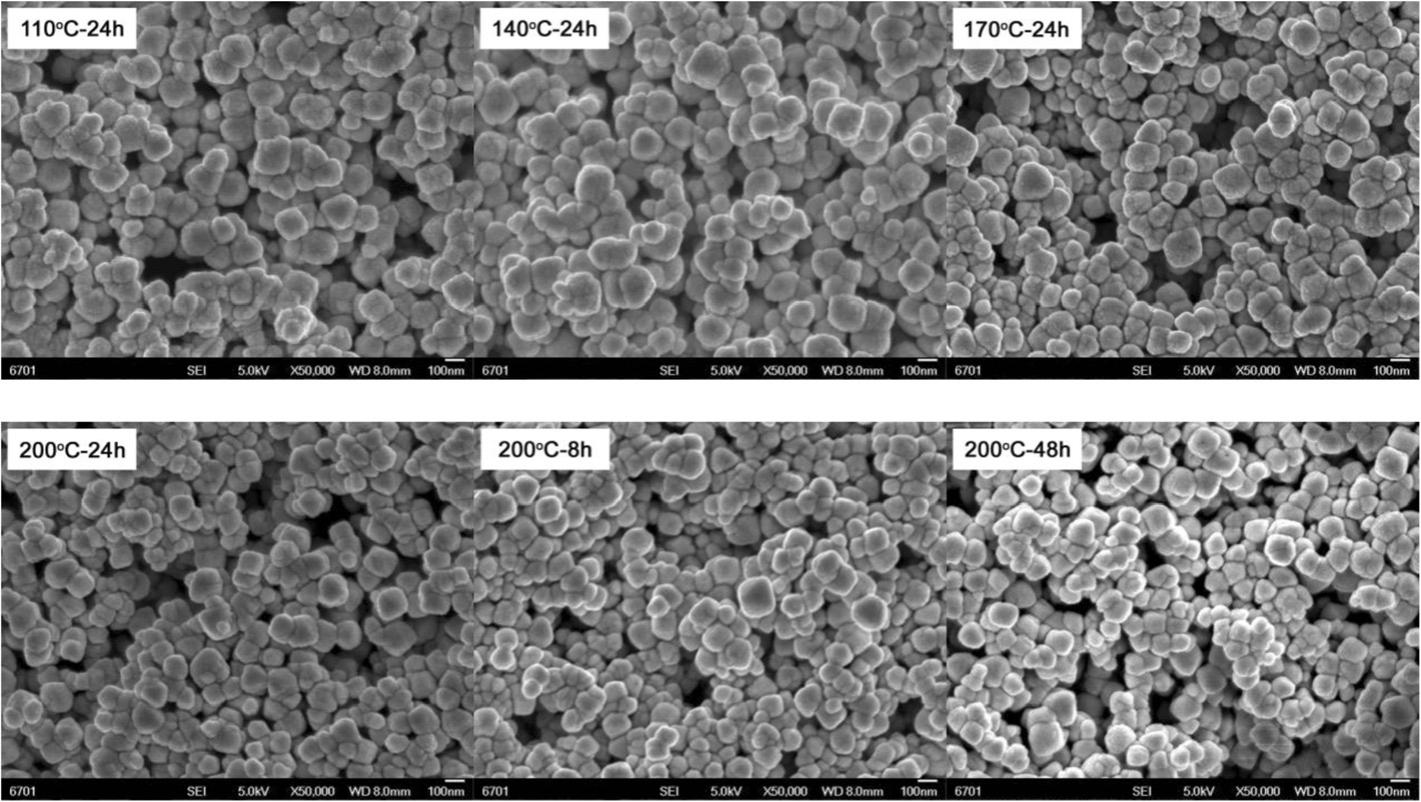
SEM images of Bi_2_WO_6_ samples prepared at different reaction temperatures and times with *C*_NaOH_ = 0.055 mol

When *C*_NaOH_ = 0.055 mol, several Bi_2_WO_6_ samples were also prepared by varying the reaction temperature from 110 to 200 °C and reaction time from 8 to 48 h. Figure 7 shows the XRD patterns of the prepared samples, revealing that all the samples crystallize in a pure Bi_2_WO_6_ phase with no traces of other impurities. Figure 8 shows the SEM images of the prepared Bi_2_WO_6_ samples. It is seen that all the samples present regular sphere-like particles without any adhesive behavior, and the average particle size is ~85 nm. This indicates that at *C*_NaOH_ = 0.055 mol, the hydrothermal reaction temperature and time have minor effect on the particle morphology and size.

### Formation Mechanism of Bi_2_WO_6_ Nanostructures

It is noted that Bi_2_WO_6_ crystals are constructed by the alternation of perovskite-like units (WO_4_)^2−^ and (Bi_2_O_2_)^2+^ layers. During the hydrothermal reaction process, Bi^3+^ ion is firstly transformed into the Bi_2_O_2_^2+^ group via its reaction with OH^−^ and H_2_O, and then, the Bi_2_O_2_^2+^ and WO_4_^2−^ ion groups assemble into Bi_2_WO_6_ nuclei, which serve as seeds for the growth of Bi_2_WO_6_ nanostructures. The nucleation and growth of Bi_2_WO_6_ nanostructures generally occurs in the region of supersaturated fluid and can be understood by the dissolution–crystallization mechanism, i.e., Ostwald ripening mechanism. The relevant chemical reactions for the crystallization of Bi_2_WO_6_ can be described as follows.

1 $$ 2{\mathrm{Bi}}^{3+} + 4{\mathrm{O}\mathrm{H}}^{\hbox{-}}\to\ {\mathrm{Bi}}_2{{\mathrm{O}}_2}^{2+} + 2{\mathrm{H}}_2\mathrm{O} $$

2 $$ 2{\mathrm{Bi}}^{3+} + 2{\mathrm{H}}_2\mathrm{O}\ \to\ {\mathrm{Bi}}_2{{\mathrm{O}}_2}^{2+} + 4{\mathrm{H}}^{+} $$

3 $$ {\mathrm{Bi}}_2{{\mathrm{O}}_2}^{2+}{{ + \mathrm{W}\mathrm{O}}_4}^{2\hbox{-}}\to\ {\mathrm{Bi}}_2{\mathrm{WO}}_6 $$

In our experiment, the NaOH content has a significant influence on the Bi_2_WO_6_ morphology, which can be interpreted by the tailoring of the crystal surface energy. At low NaOH contents of 0–0.0175 mol, the external environment has almost no effect on the surface energy of Bi_2_WO_6_ crystals, and thus, the growth of Bi_2_WO_6_ crystals is mainly controlled by the intrinsic crystal structure anisotropy. Due to its high intrinsic anisotropic property, Bi_2_WO_6_ prefers to grow into two-dimensional flake-like nanostructures. The nanoflakes have a high anisotropic surface energy and thus readily self-assemble into flower-like hierarchical microspheres. The growth and self-assembly processes take place simultaneously during the hydrothermal reaction. When the NaOH content increases up to the range of 0.03–0.0545 mol, the intrinsic anisotropic property remains largely unchanged, and as a result, Bi_2_WO_6_ still preferably grows into flake-like nanostructures. However, the surface energy anisotropy of the formed nanoflakes is significantly decreased, and thus, it cannot bring the assembly of the nanoflakes into the flower-like microspheres. Under high NaOH contents of 0.055–0.05525 mol, the surface energy of the crystals is strongly changed. On this occasion, the crystal growth is mainly controlled by the external condition and not dominant along a special direction, consequently leading to the synthesis of sphere-like Bi_2_WO_6_ particles.

### Optical Absorption Property

**Fig. 9:**
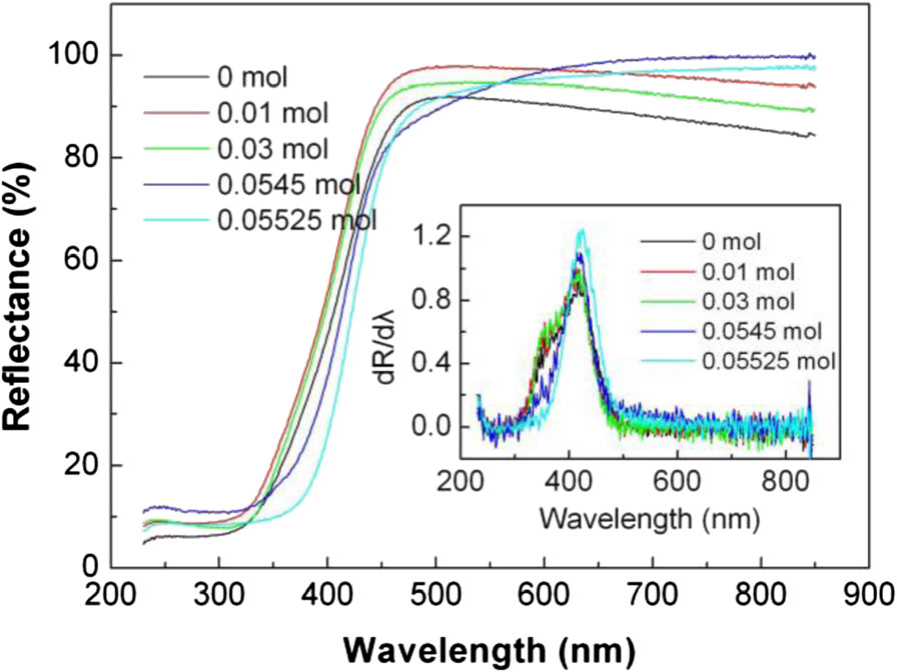
UV-visible diffuse reflectance spectra of several typical Bi_2_WO_6_ samples prepared at different NaOH contents. The *insert* shows the corresponding first derivative of the diffuse reflectance spectra

Figure 9 shows the UV-visible diffuse reflectance spectra of several typical Bi_2_WO_6_ samples prepared at different NaOH contents. The insert in Fig. 9 shows the corresponding first derivative of the reflectance (R) with respect to wavelength λ (i.e., dR/dλ). The absorption edge ascribable to the electron transition from valence band to conduction band can be determined from the peak wavelength in the first derivative spectra. The samples prepared at NaOH contents of 0–0.0545 mol (i.e., the synthesized flower-like hierarchical architectures and flake-like structures) have a similar absorption edge located at 417 nm, from which their bandgap energy *E*_g_ is obtained to be 2.97 eV. The sample prepared at the NaOH content of 0.05525 mol (i.e., the prepared sphere-like particles) has an absorption edge at 423 nm, and its bandgap energy is obtained to be 2.93 eV.

### Photocatalytic Activity

**Fig. 10:**
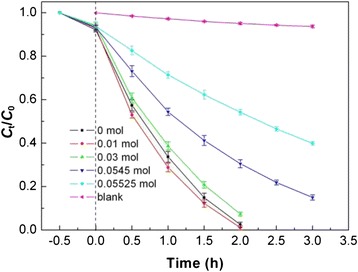
Time-dependent photocatalytic degradation of RhB over several typical Bi_2_WO_6_ samples prepared at different NaOH contents, along with the blank experiment result

Figure 10 shows the time-dependent photocatalytic degradation of RhB over several typical Bi_2_WO_6_ samples prepared at different NaOH contents under simulated-sunlight irradiation, along with the blank experiment result. The degradation percentage is defined as (*C*_0_ − *C*_t_)/*C*_0_ × 100 %, where *C*_0_ is the initial concentration of RhB and *C*_t_ is the RhB concentration after irradiation for time *t*. In the absence of the photocatalyst, RhB appears to be stable under simulated-sunlight irradiation and its degradation percentage is about 6 % after 3 h of exposure. Without simulated-sunlight irradiation, Bi_2_WO_6_ samples show a moderate adsorption toward RhB and the adsorption percentage after 0.5 h of adsorption is about 6–8 %. On irradiation with simulated sunlight and in the presence of Bi_2_WO_6_ samples, the degradation of RhB increases substantially with increasing irradiation time, implying a pronounced photocatalytic activity of as-prepared Bi_2_WO_6_ samples toward the dye degradation. Table 1 gives the degradation percentage of RhB after 2 h of photocatalysis reaction, as well as the morphology/size, BET-specific surface area, and bandgap energy of the samples. Among these samples, the samples consisting of flower-like hierarchical microspheres have relatively high photocatalytic activity. In particular, the microspheres prepared at the NaOH content of 0.01 mol exhibit the highest photocatalytic activity, and the degradation percentage reaches 98.9 % after 2 h of irradiation. It is noted that the flower-like hierarchical microspheres are constructed from thin nanosheets via the self-assembly process. The nanosheet-built flower-like hierarchical architectures can deliver a BET-specific surface area up to 72.4 m^2^ g^−1^ and expectedly provide more exposed surface active sites for the photocatalysis reaction. The lowest photocatalytic activity is observed for the sphere-like particles prepared at the NaOH content of 0.05525 mol, where the degradation percentage is about 45.8 % after irradiation for 2 h. The main reason for this is that the spherical particles have a much smaller surface area (9.5 m^2^ g^−1^) than that of thin nanosheets or nanoflakes, and less surface active sites are available for the photocatalysis reaction.

**Table 1:** Photocatalytic degradation of RhB after reaction for 2 h over Bi_2_WO_6_ samples, as well as morphology/size, BET-specific surface area, and bandgap energy of the samples

Samples	Morphology and size	*S*_BET_ (m g^−1^)	Bandgap energy (eV)	Degradation percentage (%)
*C*_NaOH_ = 0 mol (pH = 1)	Flower-like hierarchical structures,7 μm in diameter	68.9	2.97	97.5
*C*_NaOH_ = 0.01 mol (pH = 3)	Flower-like hierarchical structures,2 μm in diameter	72.4	2.97	98.9
*C*_NaOH_ = 0.03 mol (pH = 5)	Irregular flake-like structures, 55 nm in thickness	33.7	2.97	92.7
*C*_NaOH_ = 0.0545 mol (pH = 9)	Irregular flake-like structures, 60 nm in thickness	18.2	2.97	69.5
*C*_NaOH_ = 0.05525 mol (pH = 11)	Spherical structures, 85 nm in diameter	9.5	2.93	45.8

## Conclusions

The effect of NaOH content, reaction temperature, and reaction time on the hydrothermal synthesis of Bi_2_WO_6_ nanostructures was investigated. Among these experimental parameters, the NaOH content has the most important influence on the product morphology and size. Compared to the NaOH content, the reaction temperature and time has a relatively minor effect on the products. It is demonstrated that the samples prepared at *C*_NaOH_ = 0–0.0175 mol mainly consist of flower-like hierarchical microspheres, which are built up with thin nanosheets via the self-assembly process. With increasing the NaOH content, the average size of the hierarchical microspheres decreases from 7 μm at *C*_NaOH_ = 0 mol to 1.5 μm at *C*_NaOH_ = 0.0175 mol. The samples prepared at *C*_NaOH_ = 0.03–0.0545 mol exhibit irregular flake-like structures, and their size increases with the increase in NaOH content. When *C*_NaOH_ = 0.055–0.05525 mol, the prepared samples present uniform sphere-like particles with an average size of 85 nm. UV-visible diffuse reflectance spectra reveal that the samples composed of the flower-like hierarchical architectures or flake-like structures have a similar bandgap energy of 2.97 eV, while the samples presenting sphere-like particles have a bandgap energy of 2.93 eV. The photocatalytic experiments show that the as-prepared Bi_2_WO_6_ samples exhibit a pronounced photocatalytic activity toward the RhB degradation under irradiation of simulated sunlight. The highest photocatalytic activity is observed for the flower-like hierarchical microspheres prepared at *C*_NaOH_ = 0.01 mol, where the degradation percentage reaches 99 % after 2 h of irradiation.

## Abbreviations

RhB: rhodamine B
SEM: scanning electron microscope
UV: ultraviolet
XRD: X-ray powder diffraction
